# Mechanistic target of rapamycin is necessary for changes in dendritic spine morphology associated with long-term potentiation

**DOI:** 10.1186/s13041-017-0330-y

**Published:** 2017-10-30

**Authors:** Fredrick E. Henry, William Hockeimer, Alex Chen, Shreesh P. Mysore, Michael A. Sutton

**Affiliations:** 1 Neuroscience Graduate Program, University of Michigan, Ann Arbor, MI 48109 USA; 2 Molecular and Behavioral Neuroscience Institute, University of Michigan, Ann Arbor, MI 48109 USA; 3 Department of Molecular and Integrative Physiology, University of Michigan, Ann Arbor, MI 48109 USA; 4 Department of Pyschological and Brain Sciences, Johns Hopkins University, Baltimore, MD 21218 USA; 5 Molecular and Behavioral Neuroscience Institute, Department of Molecular and Integrative Physiology, University of Michigan, 5067 BSRB, 109 Zina Pitcher Place, Ann Arbor, MI 48109-2200 USA

## Abstract

Alterations in the strength of excitatory synapses in the hippocampus is believed to serve a vital function in the storage and recall of new information in the mammalian brain. These alterations involve the regulation of both functional and morphological features of dendritic spines, the principal sites of excitatory synaptic contact. New protein synthesis has been implicated extensively in the functional changes observed following long-term potentiation (LTP), and changes to spine morphology have similarly been documented extensively following synaptic potentiation. However, mechanistic links between de novo translation and the structural changes of potentiated spines are less clear. Here, we assess explicitly the potential contribution of new protein translation under control of the mechanistic target of rapamycin (mTOR) to LTP-associated changes in spine morphology. Utilizing genetic and pharmacological manipulations of mTORC1 function in combination with confocal microscopy in live dissociated hippocampal cultures, we demonstrate that chemically-induced LTP (cLTP) requires *do novo* protein synthesis and intact mTORC1 signaling. We observed a striking diversity in response properties across morphological classes, with mushroom spines displaying a particular sensitivity to altered mTORC1 signaling across varied levels of synaptic activity. Notably, while pharmacological inhibition of mTORC1 signaling significantly diminished glycine-induced changes in spine morphology, transient genetic upregulation of mTORC1 signaling was insufficient to produce spine enlargements on its own. In contrast, genetic upregulation of mTORC1 signaling promoted rapid expansion in spine head diameter when combined with otherwise sub-threshold synaptic stimulation. These results suggest that synaptic activity-derived signaling pathways act in combination with mTORC1-dependent translational control mechanisms to ultimately regulate changes in spine morphology. As several monogenic neurodevelopmental disorders with links to Autism and Intellectual Disability share a common feature of dysregulated mTORC1 signaling, further understanding of the role of this signaling pathway in regulating synapse function and morphology will be essential in the development of novel therapeutic interventions.

## Introduction

Dendritic spines comprise the primary sites of excitatory synaptic contact in the mammalian central nervous system. At mature synapses, these actin-rich protrusions are typically composed of a large head compartment densely packed with proteins of numerous types [48], and a thin neck region that attaches the head to the dendritic shaft. The high resistance of the neck can significantly boost synaptically-driven depolarization of the associated spine head [17]. The distinct structural characteristics of spines are believed to provide both chemical and electrical compartmentalization of incoming synaptic signals [5, 14].

In mature networks, synaptic connections at dendritic spines can be quite stable, as newly emergent spines generated after motor learning have been shown to persist for months [62]. Yet, individual spines have long been known to be highly dynamic structures [21]. While the distribution of spine size across the dendritic arbor of a single neuron can be quite variable [30], spine size generally correlates with excitatory synapse strength both in vitro [37] and in vivo [41]. Though clear mechanistic explanations for this correlation are just beginning to be understood [45], it is generally accepted that spine head diameter and synapse strength co-vary during the expression of long term potentiation (LTP), for example, because additional volume is required in the spine head to accommodate the insertion of additional AMPA receptors into the postsynaptic density [31, 38, 42].

The molecular mechanisms involved in the regulation of spine size and shape largely involve remodeling of the actin cytoskeleton [10]. Cytoskeletal rearrangement is necessary for the expression of long lasting plasticity at excitatory synapses, as inhibitors of actin polymerization impair LTP in the CA1 region of the hippocampus [25, 26]. The Rho family of small GTPases has been shown to play pivotal roles in the induction and maintenance of altered spine morphology, particularly in the context of long lasting synaptic plasticity [60]. An emerging model of the signaling dynamics involved in spine enlargement during LTP suggests that calcium influx through NMDARs activates CaMKIIα, leading to the subsequent recruitment of multiple RhoGTPases, wherein RhoA is critical for the initial enlargement of spine size and Cdc42 is necessary for sustaining these structural changes over time [29, 39, 40].

In addition to cytoskeletal remodeling, there is also a well-established role for new protein synthesis in the expression of long lasting plasticity at excitatory synapses. While early work focused on the contribution of cell-wide changes in gene expression via altered transcription [36], more recent evidence has established a role for de novo protein translation operating locally in dendrites during the expression of long term plasticity and memory formation [54]. Despite a clear requirement for actin remodeling as well as new protein synthesis during LTP, relatively little is known about whether these processes influence each other or are otherwise co-regulated for the expression of long lasting changes in synaptic strength.

Insofar as alterations in spine morphology during LTP are indeed bolstered by or are dependent on de novo protein synthesis, it is currently an open question as to the specific signaling pathways that may be involved in linking these processes. Given the previously demonstrated importance of BDNF signaling and protein synthesis in spine enlargement driven by local glutamate uncaging [13, 19, 58], one system of particular interest is the mechanistic target of rapamycin complex 1 (mTORC1) pathway. The mTORC1 pathway is known to be activated by BDNF signaling at excitatory synapses [49, 57] and is a well-characterized regulator of new protein synthesis, operating at the level of translation initiation [34]. mTORC1 signaling is necessary for the induction of LTP in the CA1 region of the hippocampus, where it has been demonstrated to act locally in dendrites to orchestrate the synthesis of new proteins which are crucial for long lasting changes in synaptic strength [8, 56, 61]. mTORC1 has also been shown to play a role in dendritic spine morphology as chronic pharmacological blockade of mTORC1 results in a decrease in spine density in dissociated hippocampal neurons [27]. In addition, animal models which harbor mutations leading to dysregulated mTORC1 signaling display deficits in long term potentiation [11, 53], and commonly exhibit abnormal spine morphology [28, 55]. Collectively, these results suggest that mTORC1 may play an active role in regulating new spine structure, though whether it contributes to morphological changes during long term potentiation remains an open question.

Here, we use a live cell imaging approach to demonstrate a requirement for mTORC1-dependent protein synthesis in the emergence of altered spine morphology after chemically induced LTP. We find that mTORC1 activation is not sufficient for changes in spine morphology, as transient genetic enhancement of mTORC1 activity via overexpression of a constitutively active mutant version of the upstream mTORC1 effector Rheb does not induce increases in spine head volume on its own. However, when paired with a subthreshold dose of glycine, mTORC1 activation results in robust increases in spine volume, suggesting that the combined action of mTORC1 signaling and other synaptically driven signals are required for activity-dependent changes in spine morphology. As dysregulation in spine morphology is a common feature of many neuropsychiatric disorders including autism spectrum disorders (ASD) and schizophrenia [43], a more precise understanding of the mechanisms that regulate their properties in response to changes in activity will be essential for the development of future therapeutic advances.

## Methods

### Cell culture and transfection

Dissociated postnatal hippocampal neuron cultures, prepared from postnatal day 1–2 rat pups of either sex, were plated at a density of 230–460 mm^2^ in poly-D-lysine-coated glass bottom Petri dishes (Mattek), as previously described [24]. Cultures were maintained for at least 21 DIV at 37 °C in growth medium [Neurobasal A supplemented with B27 and Glutamax-1 (Invitrogen)] before use. To achieve sparse expression, neurons were transfected with 0.5 μg of total DNA using the Ca^2+^ phosphate CalPhos Transfection kit (ClonTech) according to the manufacturer’s protocol. Unless otherwise indicated, all experiments were performed 24 h post-transfection.

### Chemically-induced LTP

Under baseline conditions, neurons were incubated in HEPES-buffered saline (HBS) containing (in mM) the following: 119 NaCl, 5 KCl, 2 CaCl_2_, 2 MgCl_2_, 30 Glucose, 10 HEPES, pH 7.4. Pharmacological induction of LTP in cultured hippocampal neurons was achieved via brief (5 min) exposure to a Mg^2+^ −free HBS solution supplemented with (in mM): 0.4 Glycine (Fisher, Waltham, MA), 0.02 Bicuculline (Tocris), and 0.003 Strychnine (Tocris, Bristol, UK) Neurons were immediately washed with warm HBS after glycine stimulation and imaged.

### Live-imaging

Neurons were imaged 1–3 days post-transfection. All imaging was performed on an inverted Olympus FV1000 laser-scanning confocal microscope using a Plan-Apochromat 63×/1.4 oil objective with 1× or 2× digital zoom. GFP was excited with the 488 nm line of an argon ion laser and emitted light was typically collected between 500 and 530 nm with a tunable emission filter. Z-stack images of eGFP signal were obtained at 10 min intervals, beginning with a pre-stimulus series of baseline measures, immediately after completion of the 5 min glycine stimulus, then regularly until 45 min post-treatment. During the imaging session, cells were perfused with HBS using a closed-loop perfusion system (Ismatec, Wertheim, Germany) and maintained at 37 °C using an in-line heater (Warner Instrument Corporation, Hamden CT). The perfusion loop was opened after stimulation to empty the system of glycine. During experiments involving treatment with anisomycin or rapamycin, these reagents were added to the HBS and perfused over the cells for the duration of the experiment.

### Analysis

Maximum projected Z-stack images (6 per cell) were first preprocessed in ImageJ (NIH, Bethesda, MD). Series of z-stacks obtained over the course of a 45 min imaging session were registered using the StackReg plugin (EPFL, Lausanne, Switzerland). Images were adjusted for size (1500 × 1500 pixels) and type (8-bit) before further processing. Automated analysis of spine morphology was performed using a custom package (‘SpineZap’) developed in the MATLAB computing environment (Mathworks, Natick, MA). Neurons were imaged so that their cell body was positioned to one corner slightly out of frame, to maximize the length of primary dendrite captured. ROI’s were defined over all visible spines on primary, secondary, and tertiary dendrites beginning immediately adjacent to the cell soma and extending progressively along the length of the primary dendrite. We did not detect obvious differences in the behavior of proximal and distal spines and those spines emanating from primary dendrites vs secondary or tertiary dendrites. The following parameters were automatically generated for each spine: head width, length, neck width and morphological class. Spines were grouped into the following morphological classes based on previously published anatomical studies using electron microscopy [15, 44]: filopodial, mushroom, flat (or “cup-shaped”) thin, and stubby. Filopodia are defined as protrusions with a length greater than or equal to 5 μm. Mushroom spines are defined as having a head to neck ratio greater than or equal to 2.5. Flat spines are defined as having a head width to length ratio greater than or equal to 1. Thin spines are defined as having a length to neck width ratio greater than or equal to 3. A spine that does not satisfy any of these conditions is classified as a stubby spine. The class of each spine is determined by checking against these conditions sequentially (in the order described above). Data analysis was performed in Origin (OriginLab, Northhampton, MA) and MATLAB. Statistical differences between multiple groups were assessed by ANOVA, followed by Tuckey’s HSD post hoc tests. For comparisons of probability distribution using the Kolmogorov–Smirnov test, alpha was set at 0.001. Ideal number of clusters for the dataset in Fig. 2e-i was determined using the NbClust package for R [9].

## Results

**Fig. 1:**
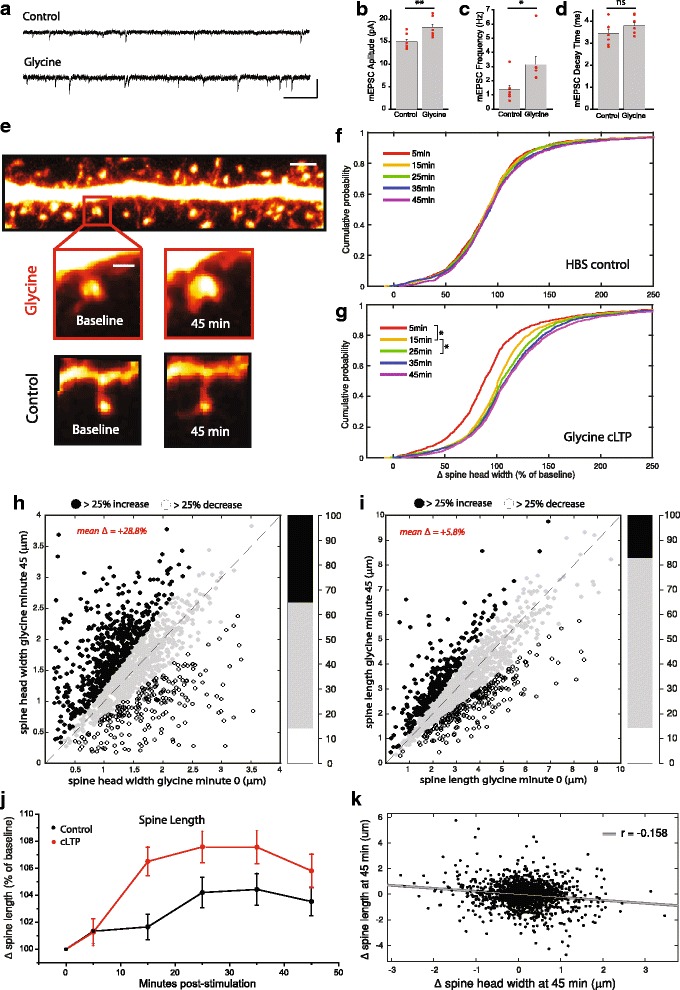
Long lasting changes in dendritic spine morphology induced by cLTP in vitro. (**a-d**) Example traces and mean (+SEM) mEPSC amplitude (**b**), frequency (**c**), and decay time (**d**) for cultured rat hippocampal neurons recorded after treatment with glycine-based cLTP (400 μM) stimulus or HBS alone as a control (*n* = 7 recordings in each condition). Glycine cLTP induces a strong increase in the strength of excitatory inputs in culture. (**e**) Example images of dendritic spines from hippocampal neurons expressing eGFP in dissociated culture under conditions of cLTP (glycine 400 μM) or HBS control. cLTP induces robust increases in dendritic spine head width. Scale bar = 2.5 μm in upper panel 1 μm in enlarged close up image. (**f-g**) Cumulative probability distributions of change in spine head diameter quantified as percent of baseline value for all spines imaged under conditions of cLTP (**g**, *n* = 1538 spines across 20 cells) or HBS alone (**f**, *n* = 1320 spines across 21 cells) at time-points 5, 15, 25, 35, and 45 m post stimulation. Glycine treatment elicits a time-dependent expansion of dendritic spine heads, * denotes significant difference in a given population from the distribution obtained in the proceeding timepoint via ks test. (**h**) Scatter plot comparing raw values of head diameter for all spines imaged before vs 45 m after treatment with 400 μM glycine during the cLTP protocol. Black symbols denote spines with head diameter increases over 25%, while white symbols mark spines that shrink by 25%. Gray symbols along the line of equality indicate spines with less extreme alterations. Bar graph on right hand side represents distribution of these groups (black = increase, white = decrease, gray = no change) as percent of total. (**i**) Comparison of pre and 45 m post stimulation values for spine length, as reported for the same set of cells in (**h**). (**j**) Timecourse of changes in spine length (represented as % of baseline values). Degree of length change was not significantly different between HBS treated controls and the glycine cLTP groups when assessed 45 m post stimulation. (**k**) Scatter plot of individual changes in protrusion length (pre vs 45 m post glycine stimulation) vs changes in head diameter for the same group of spines

To study the role of mTORC1 in spine morphological plasticity, we imaged mature cultured hippocampal neurons (> 21 DIV) transiently transfected with eGFP following cLTP induction using a glycine-induced stimulus protocol (5-min exposure to 400 μM glycine in a low Mg^2+^, HBS-based, stimulus solution). As previously reported [31, 42], we find that this induction protocol produces reliable, long-lasting increases in postsynaptic strength as assessed via changes in mEPSC amplitude and frequency in whole-cell voltage-clamp recordings (Fig. 1a-d). In cells expressing GFP to mark the extent of dendritic protrusions (Fig. 1e), we found that cLTP induction elicited a strong, time-dependent increase in spine head width, with population averages showing significant differences as early as 15 min after stimulation (Fig. 1e, Glycine group: 1538 spines across 19 neurons). Glycine treatment induced a significant rightward shift in the cumulative distribution of spine head widths in the population of assessed spines (Fig. 1g). Cells treated with HBS alone as a control group displayed no significant change from baseline levels over the course of the imaging period (Fig. 1f, Control group: 1320 spines across 24 neurons). Though we observed an average change in spine head width of +28.85% when assessed 45 min post-stimulation, the population as a whole exhibited a diverse set of responses, in terms of both valence and intensity (Fig. 1h-i). Roughly 35% of spines exhibited increases in head width over 25% of their baseline value (*n* = 543/1538), whereas 14% (*n* = 218/1538) exhibited a 25% or greater diminishment in head width. By contrast to alterations in spine head width, the changes we observed in spine length following cLTP were more subtle. While there is a trend towards spine lengthening following cLTP, the magnitude of this change is small (< 10%), and by 45 min, is not significantly different from spine length changes observed under control conditions (Fig. 1j). We also asked whether changes in spine head width and length following LTP might be related, but found no significant correlation between these morphological changes in response to glycine-induced potentiation (Fig. 1k). For these reasons, we focused our analysis primarily on spine head width in subsequent experiments.

**Fig. 2:**
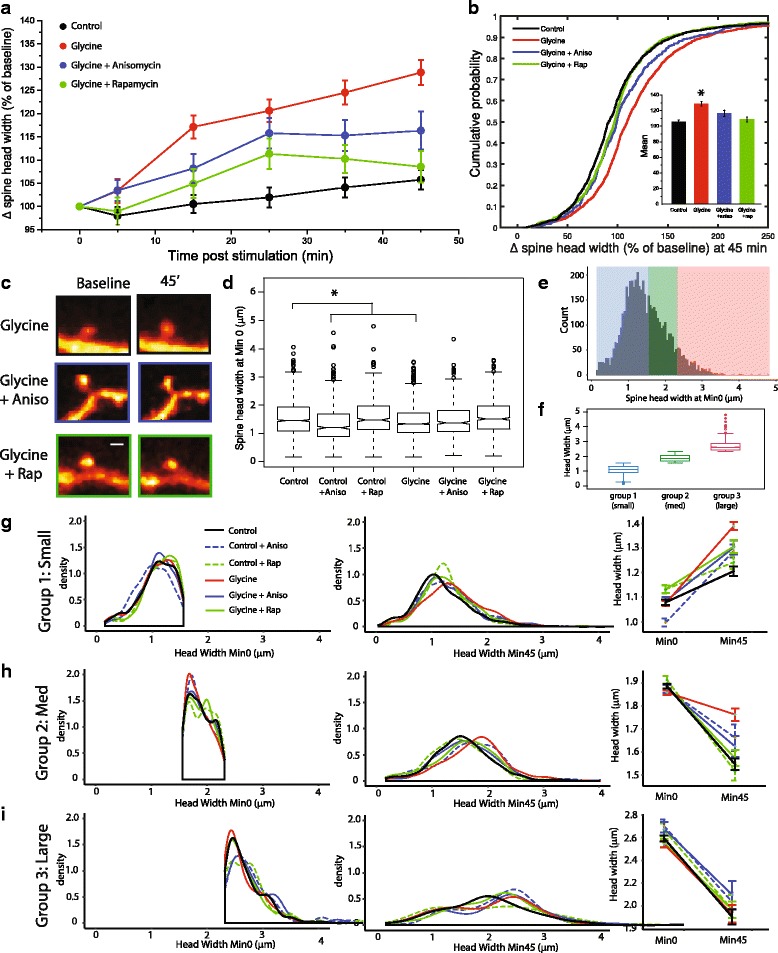
Glycine induced spine head enlargement is protein synthesis and mTORC1 dependent. (**a**) Time-course of relative changes in spine head diameter (represented as percentage of baseline value) for neurons treated with glycine (400 μM) either alone (red, n = 1538 spines across 20 cells) or after pretreatment with anisomycin (blue, *n* = 494 spines across 8 cells) or rapamycn (green, *n* = 684 spines across 9 cells). Control cells were treated with HBS alone. (**b**) Cumulative probability distribution and mean (+/−SEM) change in spine head width for cells treated with glycine with or without anisomycin, or rapamycin assessed 45 min post-stimulation. *p < 0.05 relative to control cells treated with HBS alone. (**c**) Example images taken from neurons treated with 400 μM glycine and either 40 μM anisomycin (blue) or 200 nM rapamycin (green). Scale bar = 1 μm. (**d**) Box plots showing distribution of values for spine head width across each group at baseline (recorded at min 0). One way ANOVA and post Hoc measures reveal significant variation of two groups from control values. (**e**) Frequency histogram of all spines in groups down in **d**, (*n* = 5061), colored according to groups determined by kmeans clustering. (**f**) Box plot showing range of spine head values represented in each of the three groups determined by k means cluster (Group 1 “small”, *n* = 3068; Group 2 “medium”, *n* = 1504; Group 3 “large”, *n* = 438 spines). (**g-i**) Kernel density estimates of spine head width for each cluster group as identified in 2E–F at baseline (minute 0) and 45 min post stimulus onset (middle panel). Right, direct comparison of mean values for spine head width at min0 vs min45

Having shown that structural remodeling can be reliably induced using a glycine-based stimulus protocol in mature hippocampal cultures, we next examined the role of protein synthesis in structural remodeling after chemically induced LTP (Fig. 2). As a group, glycine-treated spines exhibited a rapid enlargement in head diameter after stimulus onset, increasing roughly 17% over baseline levels by 15 min post-stimulation. Average head width in glycine-treated neurons steadily increased for the duration of the imaging session to a final value of roughly 28% larger than baseline values at 45 min post stimulation (Fig. 2a). Pre-treatment with the protein-synthesis inhibitor anisomycin (40 μM, 30 min) significantly diminished glycine-mediated increases in spine head width (Fig. 2a). This difference in mean head diameter is reflective of an overall change across the population of imaged cells, as the cumulative probability distribution of altered head diameter for spines treated with glycine + anisomycin was significantly different from the distribution of size changes in spines treated with glycine alone (Fig. 2b).

Recent work has indicated that mTORC1-mediated phosphorylation of 4E–BP is an indispensable step in the process by which this pathway controls cap-dependent translation [59]. As such, we next addressed whether our previously observed protein synthesis-dependent increases in spine head width were also dependent on mTORC1 activity. Similar to our results using anisomycin, pretreatment with the mTORC1 inhibitor rapamycin (100 nM, administered 30 min prior to glycine treatment), resulted in significantly less pronounced head enlargements than spines treated with glycine alone (Fig. 2a). By 45 min post stimulation, cells treated with glycine + rapamycin displayed a net gain in spine head width of 8.6%, which was not significantly different from HBS-treated controls (Fig. 2a). Like the effects of co-treatment with anisomycin, the cumulative probability distribution of altered head diameter for spines treated with glycine + rapamycin was significantly different from the distribution of size changes in spines treated with glycine alone (Fig. 2b). The finding that both anisomycin and rapamycin attenuate persistent increases in spine head width after glycine treatment collectively support the hypothesis that morphological plasticity after LTP relies on mTORC1-dependent protein synthesis.

We next considered the possibility that starting differences in spine size might contribute to the magnitude of relative changes in spine head area among the various treatment groups assessed above. Figure 2d shows the full distribution of spine head areas at baseline in each of the six relevant treatment conditions. Overall, the distribution of spine head widths is similar among treatments, though the proportion of large- and small-diameter spines is not uniform in all cases, which gives rise to significant differences in mean spine head widths among the conditions (ANOVA, F_5,5061_ = 18.13, *p* = 7.00e^−18^, Fig. 2d). These differences were not systematic according to pre-treatment condition, however, with only one group receiving pretreatment with anisomycin (“Control + aniso”) and one group later receiving glycine (“Glycine alone”) showing significant differences from HBS treated control values by Tukey HSD post hoc tests. Given that these baseline differences exist before treatments were initiated, it is unlikely that they reflect any specific treatment effect. However, in principle, the starting size of spines can impact the assessment of relative changes in spine size over time, as smaller spines are closer to a measurement floor and larger spines closer to a measurement ceiling. Small spines, for example, are limited in the range where they can shrink further, but there is a large dynamic range for these spines to increase in size. Accordingly, mean spine head width will tend to increase across repeated measurements even if an equal number of spines grow and shrink within a group. Likewise, for initially large diameter spines, mean spine head width will adopt a negative trajectory over time given equal proportions of spines that grow and shrink. To account for this potential issue, it was necessary to compare treatment effects on spines of different sizes separately.

To determine ideal threshold values to separate spines in our dataset according to size, we used the ‘NbClust’ package in R which compares multiple indices to determine the ideal number of naturally occurring clusters in a dataset [9]. Out of 5061 total spines across all 6 experimental conditions, we found that our data was best represented by a separation into three groups (Fig. 2e-f). Kmeans clustering revealed groups as follows: group 1 *n* = 3068 spines, range = 0.15–1.558um; group 2 *n* = 1504 spines, range = 1.559–2.317um, group 3 *n* = 489 spines, range = 2.319–4.795um (Fig. 2e-f). When partitioned into these clusters, we observed that the sampling from each group across experimental conditions was non-uniform (Control = 55.757% group 1, 32.575% group 2, and 11.666% group 3; Control + Aniso = 71.374% group 1, 17.748% group 2, and 10.877% group 3; Control + Rap = 56.28% group 1, 32.534% group 2, 11.1776% group 3; Glycine = 65.669% group 1, 27.828% group 2, 6.5019% group 3; Glycine + Aniso = 61.336% group 1, 29.959% group 2, 8.7044% group 3; Glycine + Rap = 53.070% group 1, 35.380% group 2, 11.5497% group 3). A comparison of these populations separated out by group at baseline (minute 0) or 45 min post stimulus are shown in Fig. 2g-i. We find that after clustering, within a particular spine group, mean spine head widths are now highly similar across treatment conditions at baseline (Fig. 2g-i, rightmost panels). For group 1 spines (the most represented in all treatment conditions), the glycine treatment condition exhibits an increase in spine head width that is substantively higher than in controls, despite similar spine head width in the two conditions at baseline. Likewise, for group 2 spines (the next most represented in all treatment conditions) where mean spine head width tends to decrease, the drop is noticeably less in the glycine treatment condition than in the controls. Anisomycin or rapamycin pre-treatment eliminates this effect in both cases, while having little impact when administered alone. Of note, no apparent treatment effects are evident in group 3 spines (the least represented in the data set), likely due to a measurement ceiling effect. Together, this analysis reveals that relative changes in spine head diameter are influenced by basal differences in spine head size. For the majority of spines (groups 1 and 2), glycine stimulation positively regulates spine head area relative to control conditions, an effect that is both protein synthesis- and mTORC1-dependent.

**Fig. 3:**
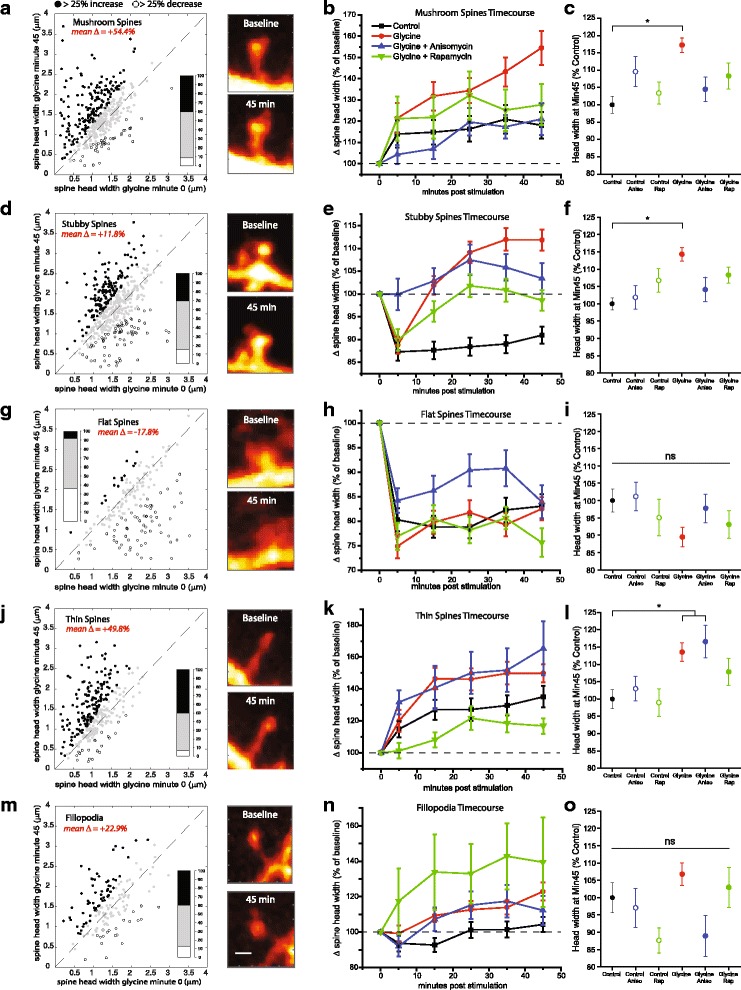
Diversity of response properties across different morphological categories during cLTP. (**a**) Scatter plot (left) and representative example images (right) of mushroom type spines (*n* = 432) comparing raw value of head diameter measured before vs 45 min after treatment with 400 μM glycine. Black symbols = > 25% increase, white symbols = >25% decrease, gray symbols = changes outside this range. Bar graph inset represents distribution of these groups as percent of total (where black = increase, white = decrease, gray = no change). (**b**) Timecourse of changes in head diameter for mushroom spines represented as percentage of baseline values after glycine treatment alone (n = 432) or in combination with anisomycin (40 μM, *n* = 119) or rapamycin (200 nM, *n* = 129), compared to HBS treated controls (*n* = 261). (**c**) Mean (+SEM) head width of mushroom spines assessed 45 min after glycine-induced potentiation with or without pretreatment from anisomycin or rapamycin. Here, all values were normalized to the average spine head width of HBS treated controls at the 45 min time point only. *p < 0.05 relative to HBS treated controls. Remaining panels as indicated above, for stubby spines (**d-f**; control stubby *n* = 487, glycine stubby *n* = 467, glycine + aniso stubby *n* = 152, glycine + rap stubby *n* = 272), flat spines (**g-i**; control flat *n* = 169, glycine flat *n* = 184, glycine + aniso flat *n* = 92, glycine + rap flat *n* = 85), thin spines (**j-l**; control thin *n* = 273, glycine thin *n* = 301, glycine + aniso thin n = 92, glycine + rap thin n = 129), and filopodia (**m-o**; control fil *n* = 125, glycine fil n = 152, glycine + aniso fil *n* = 34, glycine + rap *n* = 64). Scale bar = 1 μm

Given previous research showing unique relationships between morphological classes of dendritic spines and functional plasticity at excitatory synapses [12], we next investigated whether particular spine types display unique responsiveness to chemically-induced LTP or requirements for mTORC1 activity. Using custom Matlab analysis routines for automated morphological classification (see methods), we divided all spines from previous data sets into the following classes: mushroom (Fig. 3a-c), stubby (Fig. 3d-f), flat (Fig. 3g-i), thin (Fig. 3j-l), or filopodial (Fig. 3m-o). When parceled into these defined morphological classes, we found striking differences between spine type that were not immediately apparent from analysis of the combined group data. While we examine changes in spine width at various times following cLTP induction, we specifically focused on the 45-min post-treatment time-point for comparison between spine types. At this time-point, mushroom spines (432 of 1538 total in the glycine treatment group), exhibited an average increase in head width of 54.47% over baseline values, with 174 exhibiting increases over 25%, and 38 showing a greater than 25% decrease (Fig. 3a). Stubby spines showed a somewhat similar response pattern at this time-point, though lesser in magnitude with an average change in spine head width of 11.8% greater than baseline. Of the 468 stubby spines assessed in the glycine group, 142 displayed a > 25% increase, while 73 had >25% decrease (Fig. 3d). While thin spines (Fig. 3j) and filopodia (Fig. 3m) displayed general increases in head diameter in response to glycine treatment (Thin spines: mean 49.88% over baseline with 152/302 increasing, and 20/302 decreasing; Filopodia: mean 22.91% over baseline with 61/153 increasing and 20/153 decreasing), it should be noted that thin spines also showed a large increase in head diameter over the course of the 45 min imaging experiment under HBS control conditions as well (Fig. 3k). Flat (aka “cup-shaped”) spines showed a strikingly divergent response pattern from the other morphological classes assessed. Of the 183 total flat spines imaged in the glycine alone group, only 14 had a final change in head width over 25% of baseline values, while 67 decreased by over 25%, resulting an average change of −17.843% (Fig. 3g). However, this gradual decrease in head width was also seen in thin spines subject to HBS control solution alone (Fig. 3h), indicating that this decrement was unlikely to be due to glycine stimulation specifically.

We next assessed the requirements for protein synthesis and mTORC1 signaling during glycine-induced potentiation for each morphological sub-class. In most cases, blocking either protein synthesis (40 μM anisomycin,30 min pretreatment) or mTORC1 kinase activity (100 nM rapamycin, 30 min pre-treatment) tended to produce similar effects on changes in spine head width following cLTP induction within each sub-group. For mushroom spines (Fig. 3b-c, pre-treatment with either anisomycin or rapamycin resulted in a significant decrease in head width compared to spines treated with glycine alone (normalized control mushroom head width 45 min post stim = 100+/−40.68%, Control plus anisomycin = 109.59+/−45.4%, Control plus rapamycin = 103.39+/−36.06%, glycine = 117.27+/−43.59%, glycine + anisomycin = 104.49+/−38.77%, glycine + rapamycin = 108.32+/−43.01%; ANOVA, F_5,1181_ = 6.54, *p* = 5.22e^−6^). A similar effect was observed for stubby spines (Fig. 3e-f); normalized control stubby head width 45 min post stim = 100+/−37.42% Control + Aniso = 101.86+/−40.39%, Control + Rap = 106.74+/−41.27%, glycine = 114.29+/−41.08, glycine + anisomycin = 104.11+/−43.35%, glycine + rapamycin = 108.28+/−37.48%; ANOVA, F_5,1679_ = 6.86, *p* = 2.40e^−6^). Glycine-induced head width changes in thin spines (Fig. 3k-l) were not sensitive to protein synthesis inhibition with anisomycin (normalized control thin head width 45 min post stim = 100+/−45.71%, Control plus aniso = 102.96+/−41.77% of HBA treated controls, Control + Rapamycin = 98.97+/−41.77%, glycine = 113.56+/−47.01%, glycine + anisomycin = 116.55+/−45.49%, glycine + rapamycin = 107.86+/−44.13; ANOVA, F_5,1020_ = 4.26, *p* = 7.62e^−4^) and neither flat spines nor filopodia showed significant differences between any of the groups assessed at 45 min post-glycine stimulation (Fig. 3h-i, n-o).

**Fig. 4:**
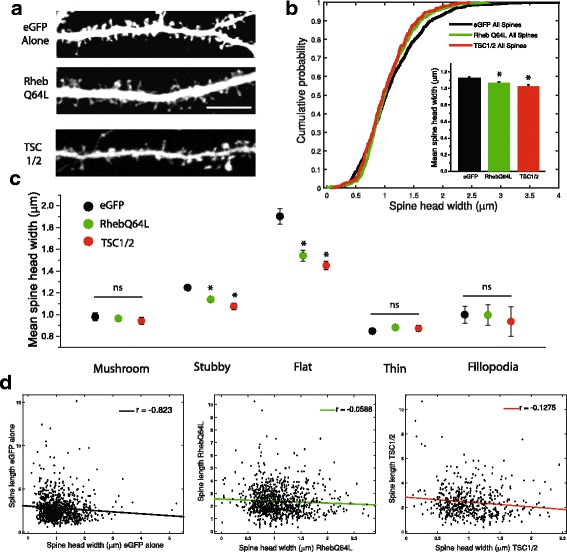
Genetic up- or down-regulation of mTORC1 activity is not sufficient to increase spine head size on its own. (**a**) Representative images of dendritic regions from hippocampal neurons 24Hrs after expression of either eGFP alone (*n* = 940 spines across 12 cells) or alongside either a constitutively active version of the positive upstream regulator RhebQ64L (*n* = 929 spines across 15 cells) or the complex of inhibitory mTORC1 effectors TSC1/2 (*n* = 509 spines across 10 cells). Scale bar =10 μm. (**b**) Cumulative probability distribution and mean (+SEM) values (inset) for spine head width in neurons with genetic alterations in mTORC1 signaling as indicated. *p < 0.05 relative to control cells expressing eGFP alone. (**c**) Mean (+/−SEM) values of spine head width in groups as indication, broken up according to morphological subtype. 24 h of genetically-mediate up- down-regulation of mTORC1 activity produced a moderate but significant decrease in dendritic head width, primarily observed in stubby and flat morphological types. *p < 0.05 relative to control cells expressing eGFP alone. (**d**) Scatter plots comparing spine head width and protrusion length for cells expression eGFP (left), RhebQ64L (middle) or TSC1/2 (right)

Collectively, these data suggest that mTORC1-dependent protein synthesis may play an important role in the maintenance of glycine-induced increases in spine head width, particularly in spines with a mushroom type morphology. In particular, it is possible that strong excitatory glutamatergic inputs interact with mTORC1 signaling to maintain altered spine morphology during long-term potentiation. As such, artificially enhanced mTORC1 signaling would predispose dendritic spines to display an enhanced response to what would otherwise be a sub-threshold excitatory stimulus. To test this directly, we transfected dissociated hippocampal neurons with RhebQ64L, a constitutively active mutant version of the GTPase that positively regulates mTORC1, to drive this signaling pathway over a period of 24 h. We have previously utilized this strategy to activate mTORC1 signaling during a similar time period, and have verified that expression of this Rheb point mutant elicits profound increases in levels of phosphorylated ribosomal protein S6, a commonly used marker of mTORC1 activity [20]. Within a population of hippocampal neurons fixed and imaged following 24 h expression of eGFP alone or alongside either RhebQ64L or the upstream mTORC1 inhibitors TSC1/2 as a basis of comparison (Fig. 4a), we observed that either genetic upregulation (RhebQ64L) or downregulation (TSC1/2) of mTORC1 activity each resulted in a slight decrease in spine head size compared to control neurons expressing eGFP alone (Fig. 4b; eGFP alone = 1.12+/−0.55um, RhebQ64L = 1.06+/−0.41um, TSC1/2 = 1.03+/−0.40um; ANOVA, F_2,2375_ = 7.16, *p* = 7.95e^−4^). This finding is perhaps surprising given previous reports of increased spine head diameter after genetic deletion of the mTORC1 inhibitor TSC1 [55], and likely reflects differences in the duration of mTORC1 activation (10–20 days in previous experiments vs 24 Hrs here). This reduction in head diameter appeared to be restricted to stubby and flat spines, as none of the other morphological subtypes displayed significant differences according to mTORC1 activation status when analyzed separately (Fig. 4c). In control neurons expressing eGFP alone, there was a small negative correlation between spine head width and overall length (Fig. 4d); this trend was unaltered following 24 Hrs of enhanced mTORC1 activity via RhebQ64L expression or down-regulation via TSC1/2, suggesting no overall change in gross spine morphology as a result of this short-term alteration in mTORC1 signaling (Fig. 4d). Taken together, these data suggest that the more broad-scale malformation in spine structure commonly associated with prolonged mTORC1 dysregulation [18, 55] does not emerge over the acute 24 h time scale used in our experiments.

**Fig. 5:**
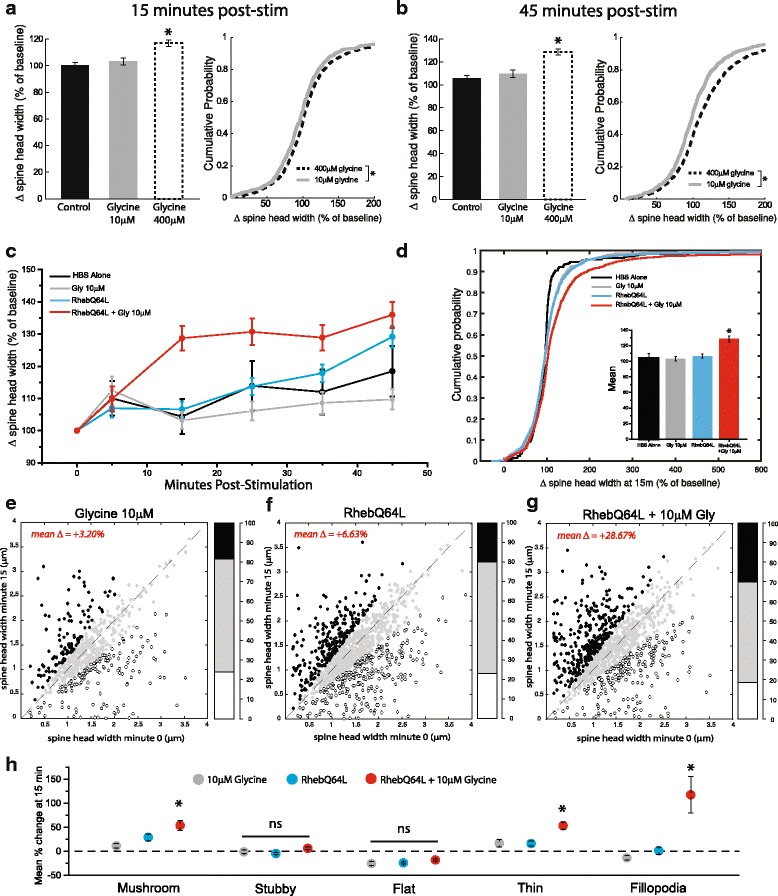
mTORC1 acts in combination with synaptically derived signals to enhance spine head diameter. (**a-b**) Mean (+/−SEM) normalized spine head width and cumulative probability distribution and of cells treated with a subthreshold (10 μM) or super-threshold (400 μM) concentration of glycine assessed 15 min (**a**) and 45 min (**b**) post-stimulation. *p < 0.05 relative to vehicle treated controls. Spines treated with a sub-threshold dose of glycine do not exhibit significant changes in head width compared to controls at either time point assessed. (**c**) Timecourse of head expansion in neurons expressing eGFP alone or in combination with genetic upregulation of mTORC1 activity via RhebQ64L expression after sub-threshold (10 μM) glycine treatment. Co-occurrence of increased mTORC1 signaling with synaptic activation elicited a rapid increase in spine head diameter observed 15 m post stimulation. (**d**) Cumulative probability distribution and mean +/−SEM values (inset) of altered spine head diameter at 15 m post stimulation shown as percentage of baseline values. HBS alone, *n* = 255 across 3 cells; 10 μM glycine, *n* = 595 across 8 cells; RhebQ64L alone, *n* = 1227 across 7 cells; RhebQ64L + 10 μM glycine, *n* = 1076 across 9 cells. (**e-g**) Scatter plots comparing raw spine head values pre vs 15 m post experiment initiation under conditions of sub-threshold glycine stimulation (**e**), expression of RhebQ64L alone (**f**), or both stimuli combined (**g**). Bar graphs on right hand side represent spines that increase (black), decrease (white) or remain stable (gray) as a proportion of total spines in each condition. Spines subject to paired activation RhebQ64L expression with sub-threshold glycine application exhibits increases in head growth well above either condition alone. (**h**) Mean (+/− SEM) of altered head diameter 15 min post stim, represented a percent change from baseline values separated according to morphological category as indicated. Rapid spine expansion after 15 m under conditions of paired synaptic stimulation and mTORC1 activation are specific to mushroom spines (10 μM glycine mushroom *n* = 185; RhebQ64L alone mushroom *n* = 336; RhebQ64L + 10 μM glycine mushroom *n* = 260), thin spines (10 μM glycine thin *n* = 57; RhebQ64L alone thin n = 169; RhebQ64L + 10 μM glycine thin n = 185), and filopodia (10 μM glycine fil *n* = 55; RhebQ64L alone fil *n* = 90; RhebQ64L + 10 μM glycine fil *n* = 53). Stubby (10 μM glycine stubby *n* = 186; RhebQ64L alone stubby *n* = 395; RhebQ64L + 10 μM glycine stubby *n* = 362) and flat spines (10 μM glycine flat n = 122; RhebQ64L alone flat *n* = 247; RhebQ64L + 10 μM glycine flat *n* = 226) show no significant differences across experimental groups

We next proceeded to ask whether constitutive mTORC1 activation may predispose spines to potentiation-like growth under conditions of sub-threshold excitatory input. We empirically determined a cLTP regimen (reducing Glycine from 400 μM to 10 μM) that produced no significant change in dendritic spine head width at any time point assessed (Fig. 5a-b). Using this sub-threshold stimulation protocol, we performed time-lapse imaging of cells expressing either RhebQ64L or eGFP alone as a control. Spine heads treated with 10 μM glycine alone (595 spines across 8 neurons) were not significantly different from vehicle treated controls at any time-point assessed after stimulation (Fig. 5c). In contrast, pairing sub-threshold glycine stimulation with persistent mTORC1 activation drove rapid spine growth (Fig. 5c), which at 15 m post-stimulation appeared similar to the increases observed following cLTP induced with 400 μM glycine at 45 m post-stimulation (Fig. 2c). These changes following RhebQ64L + 10 μM glycine persisted for at least 45 min, though the difference with the RhebQ64L alone group diminished slightly at that time-point. While, at 15 min, the mean change in spine head width for the paired RhebQ64L + 10 μM glycine group was significantly elevated compared to either condition separately or HBS controls (Fig. 5d insert; HBS control = 104.4+/−86.98% of baseline value, 10 μM glycine = 103.2+/−63.6% of baseline value, RhebQ64L alone = 106.63+/−84.1% of baseline value, RhebQ64L + 10 μM glycine = 128.75+/−122.88% of baseline value; ANOVA, F_3,3149_ = 14.09, *p* = 4.03e^−9^), a comparison of the probability distributions of the percent head width changes for these groups reveals that strong differences in these populations are carried largely by the top half of the distribution (Fig. 5d). This implies that the large increase in spine head width resulting from concurrent mTORC1 activation and low intensity stimulation with glycine was not uniform across all members of the population. Indeed, a comparison of raw values of spine head width before and 15 min post stimulus initiation reveals a significant degree of response heterogeneity across each treatment condition (Fig. 5e-g). For cells receiving sub-threshold (10 μM) glycine alone (*n* = 595 total), we observed a mean difference in spine head width of +3.203% at 15 mi post stim, with 18.5% of spines (110/595) showing increases in head width 25% over baseline values and 23.8% of spines (142/595) displaying a decrease of similar degree (Fig. 5e). Neurons expressing RhebQ64L, but receiving mock cLTP stimulation with HBS (*n* = 1227 total) showed an average change in spine head width of +6.63% when assessed 15 m after initiation of the experiment, with roughly equal numbers showing spontaneous fluctuations in head width (245 with >25% increase, 282 with >25% decrease, Fig. 5f). When genetic activation of mTORC1 activity was paired with low threshold glycine stimulation (RhebQ64L + 10 μM glycine, 1076 total), the average change in individual spine head width reflected a strong bias for potentiation, as 30% (323/1076) of spines increased in size by 25% or more and an average increase of +28.67% above baseline values was observed for the population as a whole (Fig. 5g). The heterogeneity of response properties within this population indicates that the fast alterations in spine head width might be unique to particular morphological categories. Separated out by class, we observed that spines of mushroom (10 μM glycine mushroom = 11.67+/−67.37% change from baseline, RhebQ64L mushroom = 29.22+/−129.78% change from baseline, RhebQ64L + 10 μM glycine mushroom = 53.95+/−167.94% change from baseline; ANOVA, F_2,778_ = 5.73, *p* = 0.003), thin (10 μM glycine thin = 16.87+/−89.60% change from baseline, RhebQ64L thin = 16.31+/−76.16% change from baseline, RhebQ64L + 10 μM glycine = 53.19+/−113.31% change from baseline; ANOVA, F_2,592_ = 10.57, *p* = 3.08e^−5^), and filopodial (10 μM glycine fil = −13.17+/−34.18% change from baseline, RhebQ64L fil = 1.53+/−67.10% change from baseline, RhebQ64L + 10 μM glycine = 116.12+/−267.71% change from baseline; ANOVA, F_2,195_ = 13.21, *p* = 4.17e^−6^) types were uniquely responsive to the combined effects of mTORC1 activation and low intensity glycine stimulation (Fig. 5h). Notably, we observed a diminishment in head width of flat spines over the course of imaging, in keeping with our previous observations (Fig. 3g).

**Fig. 6:**
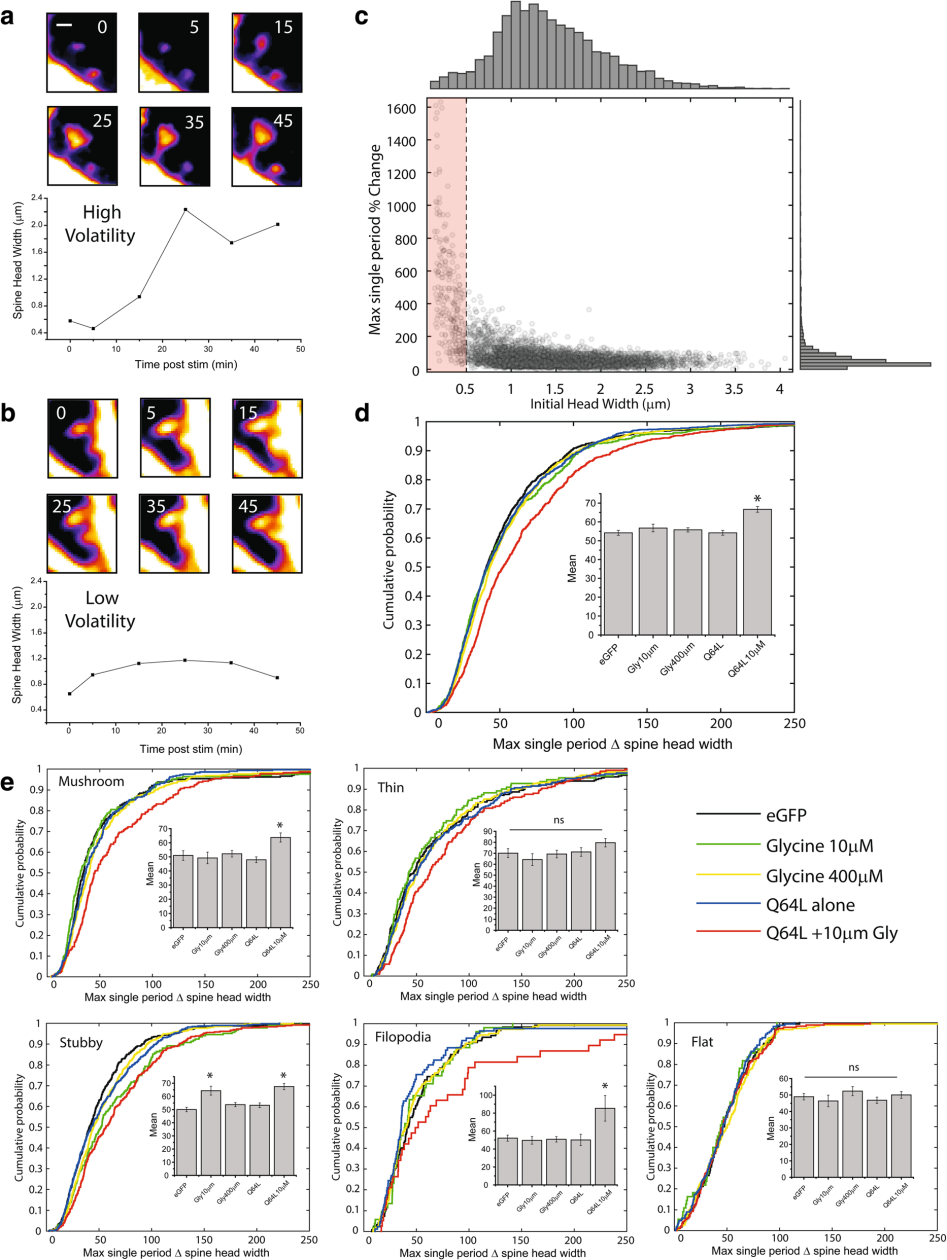
Paired mTORC1 and synaptic stimulation increases morphological volatility. (**a-b**) Representative images and example timecourses from spines sorted into categories of (**a**) “high volatility” (i.e. spines with changes spine head diameter > 200% in a single imaging timepoint) or (**b**) “low volatility” (i.e. spines that exhibit more gradual alterations in spine head diameter). Scale bar = 0.5 μm. (**c**) Scatter plot with marginal frequency histograms displaying the relationship between maximum change in head width during entire experiment (quantified as absolute percent difference from preceding timepoint) vs initial spine head diameter in μm. Each dot represents a single spine. Spines across all experimental parameters show in **d-f** included (*n* = 5756 spines). Excluding spines with head width smaller than 0.5um at minute zero (red shading) resulted in truncated data set with *n* = 5495 spines. (**d**) Cumulative probability distribution (and mean +/−SEM, inset) of the maximum single period change in head diameter (quantified as absolute percent difference from preceding timepoint) for groups as indicated. Pairing of low threshold synaptic stimulation with mTORC1 upregulation (Q64L+ 10 μM glycine) results in significantly enhanced ‘volatility’ of spine head diameter compared to all other experimental groups assessed. (ANOVA, F_4,5490_ = 13.956, *p* = 3.95e^-11^; eGFP *n* = 1267 spines, 10 μM Glycine *n* = 572 spines, 400 μM Glycine *n* = 1469 spines, RhebQ64L alone *n* = 1171 spines, RhebQ64L + 10 μM Glycine *n* = 1016 spines). * denotes significant difference from eGFP controls by Fisher LSD post hoc. (**e**) Cumulative probability distribution (and mean +/−SEM, inset) of the maximum single period change in head diameter for groups as in (**d**), separated by morphological type. * denotes significant difference from eGFP controls by Fisher LSD post hoc

When assessing individual examples of spine enlargements in the paired Rheb + glycine group, we noticed a number of spines with rapid changes in head width, often changing size to an extreme degree over the course of a single imaging time-point. An example of one of these spines can be seen in Fig. 6a. We observed many such cases, and denote this phenomenon related to sudden large changes in spine head width as “high volatility”. This feature may be distinguished from other spines whose head width may also change significantly over the course of an experiment, though at a more gradual rate of transition (denoted as “low volatility”, Fig. 6b). To explore this effect further, we examined the relationship between maximum single period (i.e. 0 to 5 min or 5 to 15 min, etc) change in head width (quantified as absolute percent difference from preceding time-point) and initial spine head diameter at min 0 (Fig. 6c). Unsurprisingly, small spines (<0.5um) tend to show the largest relative changes in head diameter compared to medium or larger sized spines. In an effort to eliminate the possibility of these smaller spines biasing our assessment of volatility differences between experimental conditions, we limited our subsequent analyses only to spines with head diameter > 0.5 μm at the start of the experiment (red box in 6C indicates excluded cases). Under these conditions, a comparison of the probability distributions (Fig. 6d) and mean value (inset) of maximum spine head change in a single time-point for each group assessed showed a significant increase in overall volatility for the paired mTORC1 activation + glycine group compared with any other condition, including the stronger glycine stimulus (400 μM) used in earlier experiments. When broken out into specific morphological subtypes, we found that mushroom spines, stubby spines and filopodia exhibited significantly enhanced volatility under conditions of paired mTORC1 upregulation and low intensity synaptic stimulation (RhebQ64L + 10 μM Glycine). We also note an unanticipated increase in volatility for stubby spines when treated with low threshold glycine (10 μM) alone. Thin and flat spine types show no significant changes between any of the groups assessed (Fig. 6e).

## Discussion

Here, we examined the role of mTORC1 signaling in structural remodeling of synapses during LTP. We show that cLTP induction caused an expansion in dendritic spine heads in cultured hippocampal neurons (Fig. 1), a specific form of structural plasticity that is widely believed to be necessary for persistent strengthening of excitatory synaptic connections. The maintenance of this altered head diameter at later time points (here, 45 m post-stimulation) is notably dependent on mTORC1-dependent protein synthesis, as it is blocked by concurrent application of anisomycin or rapamycin during glycine-induced cLTP (Fig. 2). This effect appears to be most strongly seen in a sub-population of dendritic spines with a mature, mushroom type morphology (Fig. 3). Despite being necessary to induce these changes, enhanced mTORC1 activity alone is not sufficient to elicit increased spine head diameter (Fig. 4). However, when active mTORC1 signaling is paired with a sub-threshold level of synaptic stimulation, strong changes in spine morphology rapidly emerge, resulting in significant increases in head width by 15 m post stimulation (Fig. 5). Lastly, we show that glycine-dependent changes in spine morphology occurring in the context of elevated mTORC1 signaling are highly volatile, with strong increases in head diameter often occurring between single imaging periods (Fig. 6). Collectively these results suggest that mTORC1 acts in tandem with additional activity-dependent synaptic signals to produce structural changes, and shed new light on the cellular mechanisms needed to induce structural changes underlying long lasting increases in the strength of excitatory synapses.

### A role for mTORC1-mediated protein synthesis in maintaining altered spine morphology

Of general interest was our finding that changes in spine morphology during chemically-induced LTP require cap-dependent translation. We found that pharmacological blockade of protein synthesis using anisomycin significantly diminished the extent of spine head enlargement after glycine treatment, an effect which was much stronger at 45 min post stimulation than at 15 min post stimulation (Fig. 2c). Spine head growth was similarly blocked by the mTORC1 inhibitor rapamycin (Fig. 2c-f). Given the well established role of mTORC1 in regulating translation initiation, the inhibitory effect of anisomycin/rapamycin at later time points could indicate that potentiated spines require new proteins for the maintenance of increased head width, possibly as a compensatory response to concurrent activity-dependent degradation of synaptic proteins mediated by the ubiquitin proteasome system [3, 4].

### Contribution of particular morphological types

Dendritic spines are often distinguished by morphological classification. Historically, categories have included stubby, thin, and mushroom, flat, and filopodial [15, 44], although some schemes include other categories such as bifurcated [21]. Different spine types are thought to be functionally distinct, with larger, mushroom spines widely presumed to harbor stronger synapses. It has been proposed that thin and mushroom spines contribute to learning and memory in different ways, with thin spines believed to be both more transient and plastic than mushroom spines [22]. The head diameter of thin spines is, by definition, smaller than those of mushroom spines and as such theoretically have more room to grow under LTP. For these reasons some hold that thin spines are converted into mushroom-type spines during the stabilization process of a structural engram after learning has occurred [6].

We found remarkable separation between the responsiveness of particular morphological spine classes to a glycine-based stimulation paradigm (Fig. 3). Of all types assessed, mushroom spines display particularly large changes in head diameter in response to glycine, and appear to be particularly reliant on new protein synthesis for this effect to be maintained (Fig. 2a-c). Because of their cardinal role in integrating synaptic transmission, dendritic spines are endowed with a variety of organelles and sub-cellular entities that mediate and modulate their role in synaptic plasticity. The smooth endoplasmic reticulum (SER) plays a known role in regulating calcium levels, an ion centrally implicated in LTP. Roughly half of all spines contain a SER stack [51] and the stack tends to occupy almost 20% of the total spine volume, suggesting a mechanism for regulating the calcium concentration within spines. Additionally, Ca2+ released from the SER can augment stimulus- induced Ca2+ currents [46]. It has also long been known that polyribosomes are capable of localizing to dendritic spines [52]. These dendritic polyribosomes are not uniformly distributed at synapses, but are far more prevalent in mature spines, such as mushroom spines [50], suggesting they play some specific role in mediating changes in synapse form or function during activity-induced plasticity. Furthermore, after LTP there is an increase in the proportion of spines containing polyribosomes, the presence of which can predict which spine will exhibit potentiation [33]. It will be interesting for future experiments to examine the unique mechanisms which support persistent, protein-synthesis dependent morphological changes after potentiating stimuli.

### Combinatorial action of mTOR with synaptically derived signals

Our results regarding cell autonomous mTORC1 activation paired with application of otherwise sub-threshold concentration of glycine (Fig. 5) suggest that synaptically evoked signaling mechanisms operate in conjunction with mTORC1 activity to mediate the morphological changes observed after cLTP. Constitutive mTORC1 activation alone appears to be insufficient to induce these changes (Fig. 3), possibly because they depend on synaptically driven signals to direct mTORC1-dependent translation of particular sets of mRNA to maintain altered spine structure. In such a scenario, synaptic activity would not induce a global increase in the synthesis of dendritically localized mRNAs, but might rather elicit the synthesis of specific sets of ‘LTP proteins’, a scenario that has been previously suggested for mTORC1 signaling at the synapse [1, 2]. Interestingly, we found that a sub-threshold dose of glycine can elicit rapid increases in spine size, provided this stimulus occurs on a background of high mTORC1 signaling (Fig. 5). This result supports the hypothesis that mTORC1 activation provides a context of active translation upon which even slight activity can drive synthesis of the new proteins required for changes in spine morphology. Additionally, the rapid nature of these changes is in agreement with previous reports showing an immediate impact of protein synthesis inhibitors on the extent of initial spine growth after induction of LTP at single spines using 2-photon glutamate uncaging [13, 58].

The question remains, however, as to the nature of the upstream synaptic signals that co-activate mTORC1 signaling alongside signaling pathways involved in actin cytoskeletal rearrangement. Calcium influx after LTP-inducing stimuli at single dendritic spines has been shown to induce a brief, spine-specific increase in the phosphorylation of CaMKII [29], which subsequently activates members of the Rho family of small GTPases including RhoA and Cdc42 [39, 40]. Transiently autophosphoylated CaMKII also activates the small GTPase Ras in the postsynaptic domain [16, 63]. Ras is a known activator of the PI3K/mTOR pathway [7], and it remains an intriguing possibility that Ras acts to stimulate mTORC1 directly or perhaps operates in tandem with Wnt signaling [35] to activate mTORC1 in the context of LTP. Recent work has also highlighted an important role for mTORC2 in directly regulating actin polymerization during LTP in the hippocampus [23]. This molecularly distinct complex of proteins, comprised of mTOR bound to rictor, among other partners, plays a role in regulating the actin cytoskeleton, but is usually insensitive to acute inhibition by rapamycin [32]. However, rapamycin has been shown to exert effects on mTORC2 signaling via disruption of mTOR complex formation with rictor, though only after chronic exposure of 24 h or longer [47]. As such, we find it unlikely that our reported inhibition of spine growth with acute rapamycin pre-treatment (30 min; Fig. 2) is due to an effect on mTORC2-mediated actin polymerization rather than mTORC1-mediated signaling.

Achieving a more detailed understanding of the signaling underlying structural remodeling of dendritic spines provides a window into how information is stored within the mammalian brain. Our results implicate mTORC1 activation as an important combinatorial signal that interacts with other local synaptic events to promote spine enlargement during long-lasting synaptic plasticity. Since dysregulation of mTORC1 has been strongly implicated in neurodevelopmental disorders characterized by disorders of social interaction, intellectual disability, and epileptic seizures, a better understanding of mTORC1’s role in structural plasticity may shed insight into novel therapeutic approaches for such disorders.
